# Course of Carpal Tunnel Syndrome Management in Patients With Diabetes

**DOI:** 10.1111/1753-0407.70180

**Published:** 2025-12-21

**Authors:** Sophia Xiao, Ignacio Garcia Fleury, Natalie Glass, Joseph Buckwalter

**Affiliations:** ^1^ Carver College of Medicine University of Iowa Iowa City Iowa USA; ^2^ Department of Orthopedics and Rehabilitation University of Iowa Iowa City Iowa USA

**Keywords:** carpal tunnel syndrome, diabetes complications, diabetes mellitus, diabetic neuropathies, median neuropathy

## Abstract

**Background:**

Carpal Tunnel Syndrome (CTS) is the most prevalent compressive neuropathy, often worsened by microvasculopathy in diabetic neuropathy. Diabetes mellitus (DM) patients experience increased CTS risk but are frequently underdiagnosed. This study investigates the progression to CTS diagnosis and CT release (CTR) in DM patients, aimed at better early detection and CTS prevention.

**Methods:**

Data including age and HbA1c from 304 patients with CTS, DM, and CTR (2012–2022) was collected from a tertiary care center. CTS–CTR time was compared between patients diagnosed with DM pre‐ or post‐CTS using Wilcoxon rank‐sum tests. Analyses were conducted using SAS v9.4. Relationships between age or HbA1c and CTS–CTR or DM–CTS timelines were described by Spearman correlation coefficients.

**Results:**

51% (*n* = 154) of patients received DM diagnosis post‐CTS identification. Time between diagnoses was similar in patients diagnosed with DM before vs. after CTS (24.6 (8.0–41.8), 24.3 (9.9–46.2) months, *p* = 0.604). From CTS to first CTR, the median time difference was 2.5 months (1.0–8.1) with no significant impact from DM diagnosis timing (before: 2.57, after: 2.20 months, *p* = 0.188). CTS–CTR time correlated with age (ρ = −0.24, *p* < 0.001) and HbA1c (ρ=−0.15, *p* = 0.002) at CTS diagnosis. No associations occurred with age or HbA1c at DM diagnosis (age: ρ = 0.03, *p* = 0.660, HbA1c: ρ = 0.00, *p* = 1.00).

**Conclusions:**

Over half of CTR patients were diagnosed with DM before CTS, underscoring the urgency for assessing new DM patients for CTS symptoms. Future clinical programs should target early identification of CTS signs and optimal timing for surgical interventions to enhance patient well‐being.

## Summary

- 51% of patients in this study were diagnosed with carpal tunnel syndrome before diabetes mellitus, indicating the importance of carpal tunnel screening early after new diabetes diagnoses.
- Future clinical programs should target early warning signs of carpal tunnel syndrome along with diabetes mellitus and develop standards for early screening.

## Graphical abstract

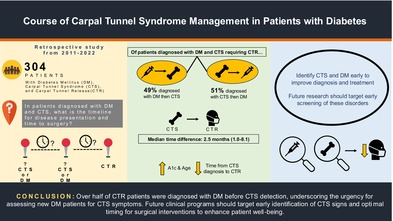

## 1 Introduction

Carpal tunnel syndrome (CTS) is the most common compressive neuropathy, occurring in 5% of the U.S. population [1], and is affected by environmental, social, and individual‐specific factors. Among patients with diabetes mellitus (DM), CTS is more common, affecting nearly 12.4 million Americans with DM per year [2, 3]. Despite the higher risk of CTS in patients with DM, it is still underdiagnosed and undertreated in this population.

Nerve compression in CTS results in a degenerative cycle of increasing pressure, blood flow obstruction, edema, and intraneural circulatory compromise. Nerve damage triggers subsequent inflammation, further aggravated by repetitive carpal movement. In this process, diabetic neuropathy is characterized by excess plasma glucose and lipids inducing metabolic alterations, resulting in increased reactive oxygen species, advanced glycation end products, ischemia, among other effects. Clinical symptoms of diabetic neuropathy associated with increased age, DM duration, and male sex [4], and are present in 30%–50% of patients with DM [5]. This population is more susceptible to CTS as well, leading to alteration in axonal transport and subsequent demyelination [5, 6, 7], compounded by the external compression of CTS. Risk factors for CTS include being age 50–59 and of female sex [8]. Patients with CTS and DM are twice as likely to have advanced disease compared to non‐DM patients [9]. Thus, understanding how to promote early detection and prevention of CTS will have a major impact on patients' treatment options, prognoses, and quality of life.

Surgical treatment of CTS following failure of non‐surgical options is standard and well‐supported, with up to 2/3 of patients receiving surgical treatment [10]. Approximately 75% of patients have their symptoms resolved after surgery [11]. While past literature has shown the benefit of carpal tunnel release (CTR) in patients with DM in restoring sensation and facilitating neurophysiological recovery [12, 13], patients with DM still have worse postoperative recovery compared to their non‐diabetic counterparts [5]. However, the temporal progression of CTS, time to surgical intervention and surgical outcomes in DM patients remain unclear. By measuring the time periods between DM diagnosis, CTS diagnosis, and CTR, this study aimed to identify the timeline from diagnosis to treatment. We hypothesized that most patients would first develop clinically recognized DM after long‐standing microvascular changes, then CTS diagnosis after compressive forces exacerbate diabetic neuropathic changes, and subsequent treatment. The evaluation of the progression of DM and CTS provides insight into the utility of early surgical intervention in promoting an effective recovery.

## 2 Methods

**FIGURE 1:**
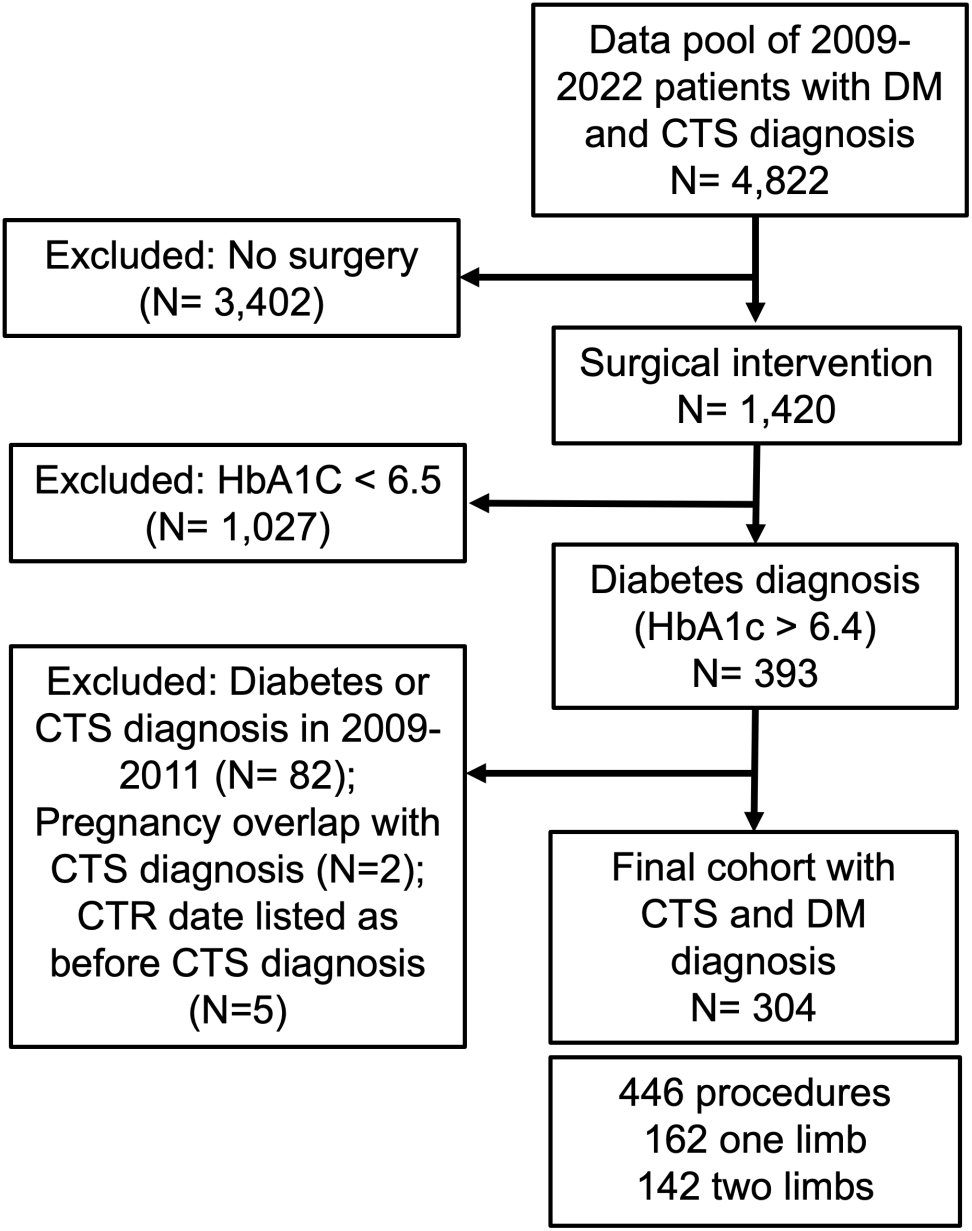
Flow chart of study selection of patients. CTS, carpal tunnel syndrome; CTR, carpal tunnel release; DM, diabetes mellitus; dx, diagnosis.

The study was reviewed and approved by the University of Iowa's Human Subjects Office (IRB# 201904825) with a waiver of informed consent. Medical record data from 4822 patients with both CTS and DM encounters at a tertiary care center in the inclusion period (2009–2022) were retrospectively identified based on diagnosis codes (DM: ICD‐9 250, ICD‐10 E08‐E11, E13; CTS: ICD‐9 354.0, ICD‐10 G56) (Figure 1). Patients with CTR (*n* = 1420) were identified and narrowed down to a final pool of 304 patients (446 limbs total, 162 single limb, 142 double limb). 1420 patients with 2295 surgeries were identified and narrowed down to only patients with DM using the American Diabetes Association criteria of HbA1C of ≥ 6.5. Transition to the institution's current electronic medical record (EMR) occurred in 2009; in order to avoid missing data or diagnoses before or during the EMR transition, patients diagnosed with either diabetes or CTS from 2009 to 2011 were excluded. Patients with concurrent pregnancy and CTS diagnosis and patients with CTR date before CTS diagnosis were removed. Patients in the final cohort all had a DM diagnosis, CTS diagnosis, and CTR; all diagnoses and surgery dates fell within the 2011–2022 study period. For patients who underwent CTR on both wrists, the first CTR was selected for analyses. Calculations of DM–CTS time period (time elapsed between DM and CTS diagnoses) assumed DM occurred first in the timeline.

Three time periods (in months) were measured: time from DM diagnosis to CTS diagnosis, time from CTS to CTR, and time from DM diagnosis to CTR. Variables like age, HbA1c, and BMI were identified at each time point. HbA1c values at 6.5 mg/dL or above were used to identify approximated DM diagnosis dates, and BMI data was taken from dates closest to or on the date of DM diagnosis. Time elapsed from CTS diagnosis to CTR was compared between patients diagnosed with DM prior to vs. after CTS using Wilcoxon rank sum tests. Spearman correlation coefficients were used to describe relationships between age or HbA1c at CTS or DM diagnosis and time from CTS to CTR or DM to CTS, respectively. Participants were divided into age groups (< 45, 45 to < 70, ≥ 70 years old) and differences between groups in CTS to CTR time were analyzed using the Kruskal–Wallis test followed by pair‐wise comparisons using Wilcoxon Rank Sum tests. *p*‐Values were adjusted for multiple comparisons using the Stepdown‐Bonferroni approach. Data were analyzed using SAS statistical software v9.4.

## 3 Results

Patient demographics can be found in Table 1. Average age, BMI, and HbA1c were identified at the time of DM diagnosis, CTS diagnosis, and CTR, with no significant difference across time periods. Average age at DM diagnosis was 57.0 ± 12.4 years, BMI 37.3 ± 9.1 kg/m^2^, and HbA1c 7.6 ± 1.6 mg/dL (Table 2).

**TABLE 1:** Baseline demographics of CTS/DM patients with CTR.

Variables	Frequency (%)
Sex	
Women	184 (61)
Men	120 (39)
Race/Ethnicity	
African American/Black	19 (6.3)
American Indian/Alaska Native	1 (0.3)
Asian	4 (1.3)
Declined	1 (0.3)
Hispanic/Latino of any race	13 (4.3)
Multiracial/Two or more races	4 (1.3)
White	262 (86.2)

**TABLE 2:** Clinical characteristics of patients at the time of DM diagnosis, CTS diagnosis, and CTR.

	Age (years)	BMI	HbA1c
DM diagnosis	57.0 ± 12.4	37.3 ± 9.1	7.7 ± 1.6
CTS diagnosis	56.9 ± 12.5	36.9 ± 8.6	7.5 ± 1.6
CTR	57.8 ± 12.7	37.1 ± 8.7	7.6 ± 1.6

*Note:* Data are presented as mean ± SD.

Abbreviations: CTR, carpal tunnel release; CTS, carpal tunnel syndrome; DM, diabetes mellitus.

**FIGURE 2:**
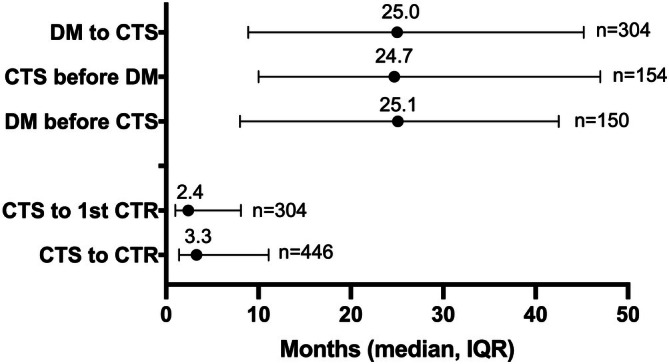
Median time periods between DM diagnosis, CTS diagnosis, and CTR dates. Figure demonstrates medians in months with lower and upper quartiles. CTS diagnosis to CTR time split into time to first CTR per patient and CTS–CTR time for each limb. CTS, carpal tunnel syndrome; CTR, carpal tunnel release; DM, diabetes mellitus; dx, diagnosis.

Two elapsed time intervals, DM–CTS and CTS–CTR, were measured. Contrary to our hypothesized time course, 51% (*n* = 154) of patients were diagnosed with DM after CTS with a median elapsed time of 24.7 [10–47] months (Figure 2). The patients diagnosed with DM before CTS had a median 25.1 [8.0–42.5] months difference. Length of time between diagnoses was similar in patients diagnosed with DM before vs. after CTS (*p* = 0.604). On average, the 304 total patients had a 25‐month DM–CTS difference. Median time from CTS to first CTR was much shorter at 2.4 [1.0–8.1] months. This did not significantly differ by timing of DM diagnosis (before: 2.57 vs. after: 2.20 months, *p* = 0.188). With all of the patients' CTRs included, the median time from CTS to any CTR was 3.3 [1.4–11.1] months.

**FIGURE 3:**
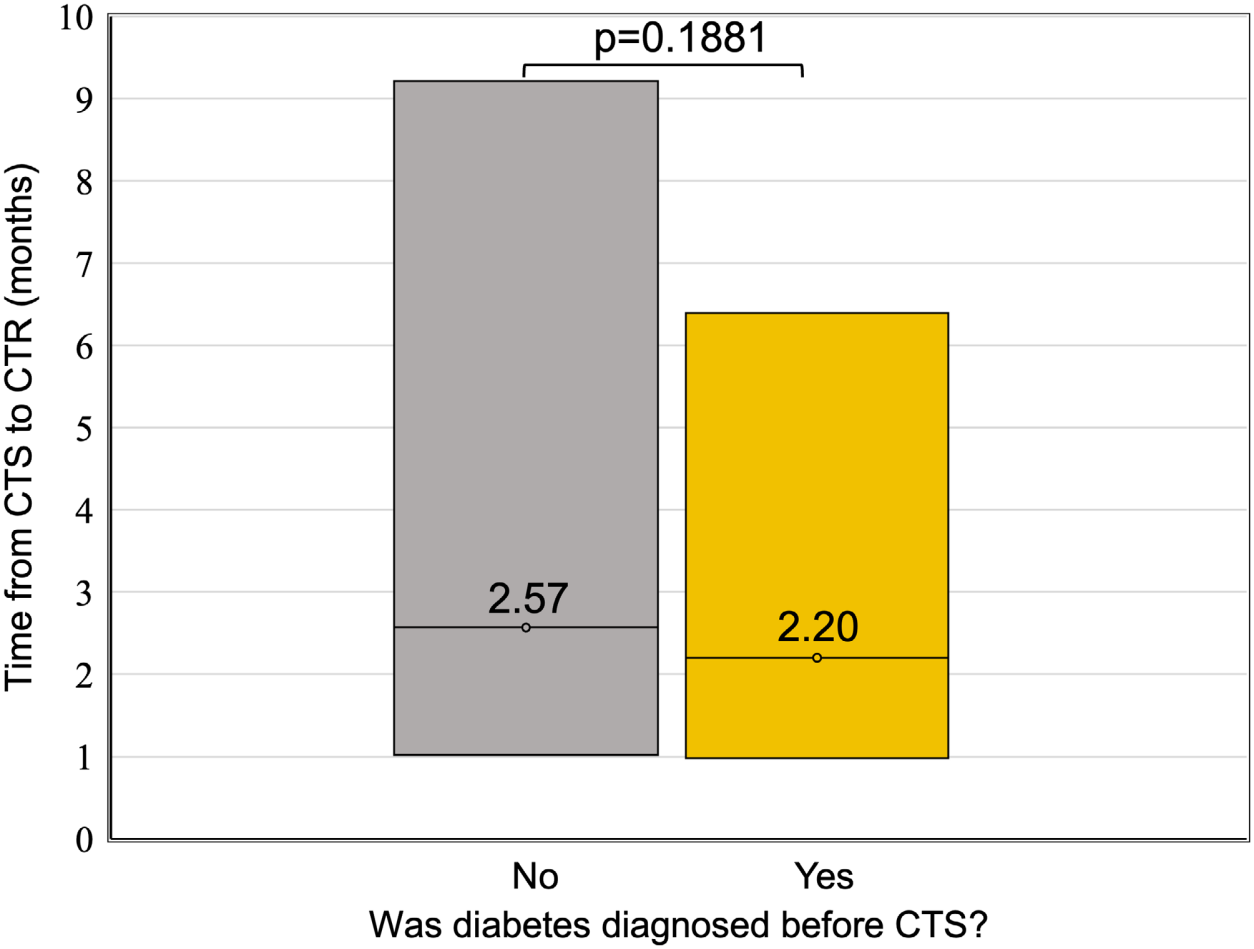
Comparison of time from CTS diagnosis to CTR between patients diagnosed with DM before and after receiving CTS diagnosis. Median values and quartile ranges in months are demonstrated (*p* = 0.1881).

We further explored the unexpected split between patients first diagnosed with DM and those diagnosed first with CTS. In comparing their CTS–CTR time, we did not identify a statistically significant time difference (*p* = 0.1881) (Figure 3).

There were small correlations between CTS–CTR time and age (*ρ* = −0.24, *p* < 0.0001) and HbA1c (*ρ* = −0.15, *p* = 0.0021) at the time of CTS diagnosis (Table 3). No significant associations were observed with age and HbA1c at DM diagnosis (age: *ρ* = 0.03, *p* = 0.660, HbA1c: *ρ* = 0.00, *p* = 1.00). An additional check was performed by grouping the ages. Patient ages 0–45 had a significantly greater median time to surgery (4.9 (IQR) months) compared to patients aged 45–70 or 70 and above (2.3 (IQR) months, *p* = 0.0051; 1.8 (IQR) months, *p* = 0.0060) (Table 4).

**TABLE 3:** Correlation of age and HbA1c with time periods between DM diagnosis/CTS diagnosis and CTS diagnosis/CTR using individual comparisons.

	DM dx to CTS dx	CTS dx to CTR
Age at DM/CTS	*ρ* = 0.03	*ρ* = −0.24
	*p* = 0.6600 (*n =* 218)	***p* < 0.0001** (*n =* 446)
A1c at DM/CTS	*ρ* = 0.00	*ρ* = −0.15
	*p* = 0.9998 (*n =* 218)	***p* = 0.0021** (*n =* 446)

*Note: p* < 0.05 are highlighted in bold font.

Abbreviations: CTR, carpal tunnel release; CTS, carpal tunnel syndrome; DM, diabetes mellitus; dx, diagnosis.

**TABLE 4:** Differences in time period from CTS diagnosis to CTR between age categories using multiple comparisons.

Group	Age (years)	*n*	Median (months)	*p*
				overall: 0.0026 (**)
1	0–45	50	4.9	1 vs. 2: 0.0051 (**)
2	45– < 70	209	2.3	1 vs. 3: 0.0060 (**)
3	≥ 70	45	1.8	2 vs. 3: 0.3400

*Note:* ** indicates *p* < 0.01.

Abbreviations: CTR, carpal tunnel release; CTS, carpal tunnel syndrome.

## 4 Discussion

This study investigated the progression of CTS and DM diagnosis to CTR. The authors originally hypothesized that in patients who will develop both DM and CTS, DM will manifest first, resulting in chronic microvasculature changes. In the setting of diabetic neuropathy, compression will then lead to CTS diagnosis with worsened presentation and treatment outcomes. However, our data demonstrated that almost all patients who underwent CTR surgery were not diagnosed with DM before CTS, disproving the original hypothesis. Approximately half the patients were diagnosed with CTS first, suggesting that often, CTS could arise before DM is detected or diagnosed. This study's findings implicate that patients who will eventually develop DM and CTS are not necessarily diagnosed with DM first, whether it is due to subclinical disease progression or inadequate screening. The data are mixed on whether a standard screening of individuals with CTS for DM would prove useful in identifying patients with possible DM. Vashishtha et al. reviewed CTS surgery patients, newly diagnosing 3 of 67 patients with DM. They concluded that not only is CTS associated with DM, but screening CTS patients is cost‐effective and useful in diagnosing existing DM [14]. Our findings support this conclusion. In contrast, De Rijk et al. concluded through their screening of CTS‐only patients that very few were positive (2/516), and therefore, systematic screening of CTS patients was not recommended for DM [15]. It should be noted that both studies used glucose blood tests as their diagnostic tool. While it has been the standard for a long time, it may be less reflective of long‐term plasma glucose and perhaps more variable [16]. Our study uses HbA1c, which was chosen for its accessibility to patients, standardization across instruments, and reliability.

This study also demonstrated that patients with DM proceed to have CTR within 2–3 months after CTS diagnosis. This finding suggests that patients with DM often require surgery very soon after diagnosis. The short time to surgery may reflect the severity of disease at presentation and implies a need to screen patients with DM for CTS earlier in order to start prevention and treatment to maximize outcomes for patients already susceptible to worse nerve recovery potential. Diabetic neuropathy and CTS can also be confidently distinguished from each other using electroneurography or ultrasound [17]. Given CTS's prevalence and that peripheral neuropathy affects up to 50% of patients with DM, an early standard screening for CTS would have wide benefits.

Additionally, this study investigated patient‐specific variables that might affect the findings, specifically age, HbA1c, and BMI. While BMI had no impact on the two time periods, age and HbA1c were both inversely associated with time to surgery. These findings are supported by past literature, which has shown that high HbA1c values, indicating worse DM control, are correlated with more residual signs of neuropathy post‐operatively [5]. Zhang et al. had shown that a 10‐year difference could increase a CTS patient's odds by 1.6 times in presenting in an advanced stage. DM could double the chance of advanced presentation [9]. In addition, variables previously shown to be associated with CTS include female gender and age 50–59 [8]. While our data had significantly fewer women than men, the average age of the patient population did fall in the demonstrated range. The explanation for this gender discrepancy is unclear as to whether fewer women are being diagnosed or if a presentation requiring surgery is more common in men.

Limitations to this study include (1) not evaluating a range of DM severity, (2) not differentiating between patients by type of DM, and (3) definition of DM diagnosis date (earliest HbA1c > 6.5 documented). After selecting for patients with DM, CTS, and CTR, the final study population had an average HbA1c of about 7.6 ± 1.6 and a BMI of approximately 37 ± 8.9, which suggests that obesity may interplay with the timing or severity of CTS and DM presentation. Patients who are obese may be more likely to have less well‐controlled DM, which in turn could impact their CTS prognosis. Patients were not differentiated by the type of DM they were diagnosed with; however, future studies could explore how the mechanistic differences in each impact time to surgery. Finally, the definition of DM used was determined using the standard American Diabetes Association criteria for HbA1c; past studies have used blood glucose and potentially used the problem list to identify diagnoses as opposed to HbA1c. Future research could investigate whether HbA1c values could be useful in identifying an early threshold (pre‐diabetic) at which to perform electroneurographic or conduction studies in identifying CTS.

Moving forward, this study's research underscores the importance of identifying CTS and DM early to improve diagnosis and treatment of these conditions. Future research should target identifying early warning signs of CTS and DM and developing standards for early screening.

## Author Contributions

S.X., I.G.F., and N.G. have full access to all study data and take responsibility for data integrity and accuracy of data analysis. S.X. designed the study, collected data, edited, reviewed, and prepared the manuscript for submission. N.G. analyzed data, edited, and reviewed the manuscript. J.B. and I.G.F. designed and supervised the study, edited, and reviewed the manuscript. All authors approved the final version of the manuscript.

## Funding

This work was supported by the National Heart, Lung, and Blood Institute, T35DK135446; University of Iowa Department of Orthopedics and Rehabilitation.

## Conflicts of Interest

The authors declare no conflicts of interest.

## Data Availability

The data that support the findings of this study are available from University of Iowa Health Care. Restrictions apply to the availability of these data, which were used under license for this study. Data are available from the author(s) with the permission of University of Iowa Health Care.

## References

[1] J. O. Sevy and M. Varacallo , “Carpal Tunnel Syndrome,” in StatPearls (StatPearls Publishing, 2023), accessed October 21, 2023, http://www.ncbi.nlm.nih.gov/books/NBK448179/. 28846321

[2] CDC , The Facts, Stats, and Impacts of Diabetes. Centers for Disease Control and Prevention. Published April 4, 2023, accessed October 21, 2023, https://www.cdc.gov/diabetes/library/spotlights/diabetes‐facts‐stats.html.

[3] A. Vinik , A. Mehrabyan , L. Colen , and A. Boulton , “Focal Entrapment Neuropathies in Diabetes,” Diabetes Care 27, no. 7 (2004): 1783–1788, 10.2337/diacare.27.7.1783. 15220266

[4] Factors in development of diabetic neuropathy , “Baseline Analysis of Neuropathy in Feasibility Phase of Diabetes Control and Complications Trial (DCCT). The DCCT Research Group,” Diabetes 37, no. 4 (1988): 476–481. 2897940

[5] M. Zimmerman , A. Gottsäter , and L. B. Dahlin , “Carpal Tunnel Syndrome and Diabetes—A Comprehensive Review,” Journal of Clinical Medicine 11, no. 6 (2022): 1674, 10.3390/jcm11061674. 35329999 PMC8952414

[6] Pathogenesis of Diabetic Polyneuropathy—UpToDate, accessed October 21, 2023, https://www‐uptodate‐com.proxy.lib.uiowa.edu/contents/pathogenesis‐of‐diabetic‐polyneuropathy.

[7] E. Rota and N. Morelli , “Entrapment Neuropathies in Diabetes Mellitus,” World Journal of Diabetes 7, no. 17 (2016): 342–353, 10.4239/wjd.v7.i17.342. 27660694 PMC5027001

[8] J. Low , A. Kong , G. Castro , P. Rodriguez de la Vega , J. Lozano , and M. Varella , “Association Between Diabetes Mellitus and Carpal Tunnel Syndrome: Results From the United States National Ambulatory Medical Care Survey,” Cureus 13, no. 3 (2021): e13844, 10.7759/cureus.13844. 33859898 PMC8038929

[9] D. Zhang , J. Collins , P. Blazar , and B. E. Earp , “Factors Associated With Advanced Presentation for Carpal Tunnel Release,” Journal of Hand Surgery 45, no. 2 (2020): 111–116, 10.1016/j.jhsa.2019.08.016. 31668408

[10] S. Hulkkonen , K. Lampainen , J. Auvinen , J. Miettunen , J. Karppinen , and J. Ryhänen , “Incidence and Operations of Median, Ulnar and Radial Entrapment Neuropathies in Finland: A Nationwide Register Study,” Journal of Hand Surgery, European Volume 45, no. 3 (2020): 226–230, 10.1177/1753193419886741. 31739732

[11] J. D. P. Bland , “Treatment of Carpal Tunnel Syndrome,” Muscle & Nerve 36, no. 2 (2007): 167–171, 10.1002/mus.20802. 17534984

[12] N. O. B. Thomsen , G. S. Andersson , J. Björk , and L. B. Dahlin , “Neurophysiological Recovery 5 Years After Carpal Tunnel Release in Patients With Diabetes,” Muscle & Nerve 56, no. 6 (2017): E59–E64, 10.1002/mus.25633. 28241376

[13] Y. Tu , W. C. Lineaweaver , Z. Chen , J. Hu , F. Mullins , and F. Zhang , “Surgical Decompression in the Treatment of Diabetic Peripheral Neuropathy: A Systematic Review and Meta‐Analysis,” Journal of Reconstructive Microsurgery 33, no. 3 (2017): 151–157, 10.1055/s-0036-1594300. 27894152

[14] M. Vashishtha , B. Varghese , F. Mosley , A. Kadakia , and W. de Jager , “Screening for Thyroid Dysfunction and Diabetes in Patients With Carpal Tunnel Syndrome,” Surgeon 14, no. 3 (2016): 147–149, 10.1016/j.surge.2014.11.003. 25533047

[15] M. C. de Rijk , F. H. Vermeij , M. Suntjens , and P. A. van Doorn , “Does a Carpal Tunnel Syndrome Predict an Underlying Disease?,” Journal of Neurology, Neurosurgery, and Psychiatry 78, no. 6 (2007): 635–637, 10.1136/jnnp.2006.102145. 17056628 PMC2077979

[16] D. B. Sacks , “A1C Versus Glucose Testing: A Comparison,” Diabetes Care 34, no. 2 (2011): 518–523, 10.2337/dc10-1546. 21270207 PMC3024379

[17] N. C. Drăghici , M. M. Tămaș , D. C. Leucuța , et al., “Diagnosis Accuracy of Carpal Tunnel Syndrome in Diabetic Neuropathy,” Medicina (Kaunas, Lithuania) 56, no. 6 (2020): 279, 10.3390/medicina56060279. 32517033 PMC7353862

